# STAT6 inhibition of M2 macrophages suppresses tumor growth by modulating the tumor microenvironment in colon cancer model

**DOI:** 10.3389/fimmu.2026.1733991

**Published:** 2026-06-03

**Authors:** Geumseong Yeun, Ik-Hwan Han, Ilseob Choi, Soyoung Kim, Hyojung Choi, Hyunsu Bae

**Affiliations:** 1 Department of Science in Korean Medicine, College of Korean Medicine, Kyung Hee University, Seoul, Republic of Korea; 2 Department of Physiology, College of Korean Medicine, Kyung Hee University, Seoul, Republic of Korea; 3 Twinpig Biolab Inc. R&D Center, Seoul, Republic of Korea; 4 Department of Korean Medicine, Graduate School, Kyung Hee University, Seoul, Republic of Korea

**Keywords:** colorectal cancer, immunotherapy, peptide-drug conjugate, STAT6, tumor microenvironment, tumor-associated macrophages

## Abstract

**Background:**

Colorectal cancer (CRC) progression is strongly influenced by the tumor microenvironment (TME), where M2 tumor-associated macrophages (TAMs) establish an immunosuppressive milieu that promotes tumor growth, angiogenesis, and immune evasion. Targeting M2 TAMs has therefore emerged as a promising strategy to overcome therapeutic resistance and restore antitumor immunity. Here, we examined the effect of STAT6 modulation in M2 macrophages on reprogramming the tumor microenvironment and enhancing antitumor immune responses in colorectal cancer.

**Methods:**

We developed a peptide drug conjugate, TAMpep-IP, composed of an M2-homing peptide (TAMpep) and a STAT6-inhibitory peptide (IP), designed to selectively inhibit STAT6 signaling in M2 TAMs. The inhibitory effects of TAMpep-IP on STAT6 phosphorylation and M2-associated markers were evaluated in THP-1-derived macrophages. Antitumor efficacy of TAMpep-IP was assessed in a CT26 murine colon cancer model through analyses of tumor growth, macrophage phenotype, and T-cell activation.

**Results:**

TAMpep-IP effectively reduced STAT6 phosphorylation and downregulated M2-associated genes and proteins, including CD206, Arg-1, TGF-β, and IL-13, while upregulating the pro-inflammatory cytokine IL-1β. *In vivo*, TAMpep-IP treatment significantly suppressed tumor growth and proliferation, accompanied by a marked reduction of M2-like TAM infiltration and decreased TGFB expression in tumor tissues. Importantly, TAMpep-IP enhanced antitumor immunity by increasing the population of Granzyme B^+^ CD8^+^ T cells and reducing PD-1^+^ and TIM-3^+^ exhausted CD8^+^ T cells, along with elevated levels of TNF-α, IL-1β, and IL-12.

**Conclusion:**

These findings demonstrate that TAMpep-IP reprograms the immunosuppressive TME by selectively suppressing STAT6 signaling in M2 TAMs, thereby restoring cytotoxic T-cell activity and inhibiting tumor progression. This selective strategy offers a promising therapeutic avenue to overcome TAM-mediated immune suppression and enhance the efficacy of existing immunotherapies.

## Introduction

Colorectal cancer (CRC) is one of the most prevalent and lethal gastrointestinal malignancies worldwide, accounting for approximately 1.9 million new cases and over 900, 000 deaths annually (1, 2). Its incidence continues to rise due to aging populations, dietary habits, and chronic inflammation, reflecting a complex interplay of genetic, environmental, and immunological factors (3). Despite significant advances in surgery, chemotherapy, and immunotherapy, treatment outcomes remain limited, largely owing to tumor heterogeneity and immune evasion (4, 5). These challenges underscore the need for therapeutic approaches that address the immunological complexity of CRC rather than targeting cancer cells (6).

Recent studies have highlighted the pivotal role of the tumor microenvironment (TME), particularly immune and stromal interactions, in shaping CRC progression, metastasis, and therapeutic resistance (7–9). In CRC, durable therapeutic benefit is often constrained by an immunosuppressive TME that suppresses antitumor immunity, facilitates immune evasion, and promotes angiogenesis and metastasis, ultimately contributing to poor clinical outcomes (10, 11). Therefore, reprogramming the TME toward a pro-inflammatory, tumor-inhibitory state has emerged as a promising strategy to enhance immunotherapeutic responsiveness (12, 13).

Among the diverse immune components of the TME, tumor-associated macrophages (TAMs) represent a dominant population influencing progression of CRC (2, 14). TAMs exhibit remarkable functional plasticity and can polarize into classically activated M1 macrophages, which produce pro-inflammatory cytokines (IL-1β, TNF-α) and mediate antitumor activity, or alternatively activated M2 macrophages, which secrete immunosuppressive mediators such as TGF-β and Arg-1 that promote tumor growth, angiogenesis, and metastasis (15). Accumulating evidence shows that M2 TAMs directly impair CD8^+^ T-cell activation and effector function by limiting Granzyme B production and sustaining exhausted phenotypes characterized by PD-1 or TIM-3 expression (16, 17). High densities of M2 TAMs are therefore associated with reduced T-cell infiltration, immune checkpoint resistance, and poor prognosis in CRC (17, 18). Conversely, therapeutic strategies that reprogram M2 TAMs toward a pro-inflammatory phenotype have been shown to enhance CD8^+^ T-cell cytotoxicity and restore antitumor immunity (19).

The JAK/STAT signaling pathway, particularly signal transducer and activator of transcription 6 (STAT6), is a pivotal regulator of macrophage polarization and immune homeostasis within the tumor microenvironment (20, 21). Upon stimulation by IL-4 and IL-13, STAT6 becomes phosphorylated and translocated to the nucleus, where it induces transcription of M2-associated genes such as Arg-1, CD206 (MRC1), TGF-β, and IL-10, thereby promoting an immunosuppressive phenotype that facilitates tissue remodeling, angiogenesis, and tumor progression (22). In CRC, elevated STAT6 activation in TAMs has been correlated with increased M2 macrophage infiltration, enhanced tumor growth, and poor patient prognosis (23). Conversely, inhibition of STAT6 signaling suppresses the expression of M2-related cytokines and surface markers while restoring pro-inflammatory mediators such as IL-1β, TNF-α, and IL-12, shifting the macrophage phenotype toward an M1-like, tumoricidal state (24). Therefore, targeting STAT6-driven M2 polarization may represent a selective therapeutic strategy to reprogram the immunosuppressive TME and enhance antitumor immune responses in CRC.

In this study, we aimed to investigate whether selective inhibition of STAT6 in M2 TAMs could remodel the immunosuppressive TME and enhance antitumor immunity in CRC. We synthesized a novel peptide-drug conjugate (TAMpep-IP) by conjugating a STAT6 inhibitory peptide (STAT6-IP), which binds to the phosphorylation site of Tyr641 on STAT6 and thereby inhibits its activation and downstream transcriptional signaling (25, 26), with TAMpep, a peptide that selectively binds to M2-like TAMs (27). We then evaluated whether TAMpep-IP could inhibit M2 polarization and enhance CD8^+^ T-cell activation while reducing exhausted CD8^+^ T cells in TME of CRC model.

## Materials and methods

### Peptide synthesis

Peptides used in this study were synthesized by GenScript (Piscataway, New Jersey, USA): TAMpep (PEG2-VLTTGLPALISWIKRKRQQ), IP (RGYVPAT), and TAMpep-IP (PEG2-VLTTGLPALISWIKRKRQQ-G-RGYVPAT). All peptides were purified to greater than 95% purity. The peptides were dissolved in sterile distilled water containing 0.1% acetic acid and diluted in DPBS (Welgene, Gyeongbuk, Republic of Korea).

### Cell culture

The human monocytic leukemia cell line THP-1 (KCLB; Korean Cell Line Bank, Seoul, Korea) was cultured in RPMI 1640 medium (Welgene, Gyeongsangbuk, Korea) supplemented with 10% heat-inactivated fetal bovine serum (FBS) and 1% penicillin–streptomycin (Hyclone, Logan, UT, USA). Cells were subcultured every 2–3 days upon reaching approximately 80% confluence and maintained at 37 °C in a humidified incubator with 5% CO_2_ for all experiments.

The murine colon carcinoma cell line CT26 (KCLB 80009; Korean Cell Line Bank, Seoul, Korea) was cultured in Dulbecco’s modified Eagle’s medium (DMEM) (D5671; Welgene, Gyeongsangbuk, Korea) supplemented with 10% heat-inactivated FBS, 100 U/mL penicillin, and 1% penicillin–streptomycin. Mouse macrophage cell line RAW264.7 cells were purchased from the American Type Culture Collection (Rockville, MD). Cells were cultured in DMEM medium with 10% heat-inactivated FBS, 100 U/mL penicillin, and 1% penicillin–streptomycin. Cells were incubated under the same conditions as above.

### Macrophage polarization

THP-1 monocytes were differentiated into macrophages by treating cells with 100 nM phorbol 12-myristate 13-acetate (PMA; Sigma-Aldrich) for 24 h. Cells were then incubated in RPMI1640 containing 10% FBS for 48h for non-polarized M0 macrophages. To induce M1 polarization, cells were stimulated with 100 ng/mL lipopolysaccharide (LPS; Sigma Aldrich) and 20 ng/mL recombinant human interferon (rhIFN)-γ (Prospec, Ness Ziona, Israel). M2 macrophage polarization was induced by treating cells with 20 ng/mL recombinant human interleukin-4 (rhIL-4; PeproTech) and 20 ng/mL rhIL-13 (PeproTech) for 24 h. Raw264.7 macrohpages were polarized into M2 macrophages by treated with 10ng.ml recombinant murine interleukin-4 (IL-4; Miltenyi Biotec) and 10 ng/mL recombinant murine interleukin-13 (IL-13; Sigma-Aldrich) for 24 h.

### Tumor inoculation and animal study

BALB/c female mice (6–8 weeks old) were purchased from DBL (Chungbuk, Korea). Animal procedures were approved by the University of Kyung Hee Institutional Animal Care and Usage Committee (KHSASP-25-148). After a one-week adaptation period, a subcutaneous tumor model was established by injecting CT26 tumor cells (3 × 10^5^ cells) into the right flank of each mouse. The mice were then maintained for an additional week to allow tumor establishment. To evaluate the therapeutic effect of TAMpep-IP, the mice were randomly divided into two groups: a control group (n = 6) and a TAMpep-IP group (n = 6). TAMpep-IP was dissolved and diluted in PBS immediaterly before administration and injected subcutaneously every three days for a total of seven injections. The control group recevied PBS alone and was used as the vehicle control group. Tumor size was measured using a digital caliper. Tumor volume was calculated using the following formula: (width × width × length)/2. Following the final dose, the mice were sacrificed, and tumor tissues were collected for further analysis. Mice were anesthetized with isoflurane and euthanized. Tumor tissues were subsequently harvested using sterile surgical instruments.

### Real-time PCR

Total RNA was extracted from cells and mouse tumor tissues using the easy-BLUE RNA extraction kit (iNtRON Biotechnology, Seongnam, Korea), according to the manufacturer’s instructions. The cDNA was synthesized using the Maxime RT PreMix Kit (iNtRON Biotechnology). Real-time PCR was performed using a CFX Connect System (Bio-Rad Laboratories, Hercules, CA, USA). The expression levels of the target mRNAs were normalized to the expression levels of GAPDH, a housekeeping gene. Each reaction was performed in triplicate. The cycling conditions were as follows: initial denaturation at 95 °C for 3 minutes, followed by 39 cycles of denaturation at 95 °C for 10 seconds, annealing at 55 °C for 10 seconds, and extension at 72 °C for 30 seconds with fluorescence acquisition. After amplification, a melt curve analysis was performed from 65 °C to 95 °C with 0.5 °C increments every 5 seconds. The primer sequences are shown in **Supplementary Tables 1** and **2**.

### Western blotting

Total protein was extracted from cells and mouse tumor tissues using PRO-PREP™ Protein Extraction Solution (iNtRON Biotechnology, Seongnam, Korea). The protein was quantified using the Pierce™ BCA Protein Assay Kit (Thermo Fisher Scientific, Waltham, MA, USA). The protein was separated on 10% SDS–polyacrylamide gels and transferred to nitrocellulose membranes using the Trans-Blot Turbo Transfer System (Bio-Rad, Hercules, CA, USA). Membranes were blocked with 5% bovine serum albumin (BSA) in TBS-T(20 mM Tris-HCl, pH 7.4; 150 mM NaCl; 0.05% Tween-20) and incubated overnight at 4 °C with the following primary antibodies: STAT6 (1:1000, #9362S; Cell Signaling Technology, Danvers, MA, USA), Phospho-STAT6 (p-STAT6) (1:1000, #56554S; Cell Signaling Technology), CD86 (1:1000, #19589S; Cell Signaling Technology), CD206 (1:1000, #ab64693; Abcam, Cambridge, UK), β-actin (1:40, 000, #AC038; ABclonal, Woburn, MA, USA) After washing with TBS-T buffer (20 mM Tris-HCl, pH 7.4; 150 mM NaCl; 0.05% Tween-20), membranes were incubated with horseradish peroxidase (HRP)-conjugated secondary antibodies for 1 hour at room temperature. Protein bands were detected using the D-Plus™ ECL Femto System (Donginbiotech Co., Ltd., Seoul, Korea) and quantified using ImageJ software (NIH, Bethesda, MD, USA).

### Flow cytometry analysis

Tumor tissues were enzymatically dissociated in serum-free medium containing collagenase D (1 mg/mL; Sigma-Aldrich) and DNase I (1 mg/mL; Sigma-Aldrich) at 37 °C for 15 minutes using a shaking incubator. The tissues were dissociated using a MACS dissociator and MACS C tube (Miltenyi Biotec, Auburn, CA, USA). The tissues were then filtered using a 40-μm cell strainer (Corning Incorporated, Corning, NY, USA) to obtain single-cell suspensions. Red blood cells were lysed using 1× RBC lysis buffer (Invitrogen, Carlsbad, CA, USA) for 5 minutes at room temperature. After washing, cells were resuspended in staining buffer (BD Biosciences, San Jose, CA, USA) and used for surface and intracellular staining. For flow cytometry analysis, the following antibodies were purchased from BD Biosciences or Biolegend (San Diego, CA, USA) and used as indicated For identification of macrophages, the following antibodies (BD Biosciences) were used: CD45–FITC, CD11b–PerCP-Cy5.5, F4/80–BV421, CD86–BV786, CD206–APC For analysis of tumor-infiltrating T cells, the following antibodies (BD Biosciences) were used: CD45–APC-Cy7, CD8–PE-Cy7, Granzyme B–FITC, TIM3–PerCP-Cy5.5. For detection of phosphorylated STAT6, cells were stained with Brilliant Violet 421™ anti-STAT6 (pTyr641) antibody (#686019; BioLegend, San Diego, CA, USA). All antibodies were incubated with cells for 30 minutes in staining buffer. For intracellular staining, cells were treated with 1× Fixation/Permeabilization Buffer (BD Biosciences) for 30 minutes at room temperature. Flow cytometry was performed using a FACSLyric™ cytometer (BD Biosciences).

### ELISA assay

To assess TGF-β1 and IL-13 levels, THP-1 cells were polarized into M2 macrophages using the aforementioned methods. M2-polarized macrophages were treated with TAMpep-IP and incubated for 72 h. The culture supernatants were then collected, and cytokine concentrations were measured using the Mouse TGF-β1 DuoSet ELISA kit (DY240) and Mouse IL-13 DuoSet ELISA kit (DY213) (both from R&D Systems, Minneapolis, MN, USA). ELISA was performed according to the manufacturer’s instructions.

### Immunofluorescence analysis and immunohistochemistry analysis

THP-1 cells were seeded at a density of 5 × 10^4^ cells/well on chamber slide 4 well (Thermo Fisher Scientific, Waltham, MA, USA) and were polarized into M0, M1, M2 macrophages, and TAMs using the methods described above. The cells were washed, fixed with 4% paraformaldehyde for 15 minutes at room temperature, and blocked with 5% bovine serum albumin (BSA) for 1 h. The chamber slide was then incubated with Phospho-STAT6 (p-STAT6) (1:200, #56554S; Cell Signaling Technology), and 4’, 6-diamidino-2-phenylindole (DAPI) (Vector Laboratories, Burlingame, CA, USA) to visualize the nuclei. All images were captured using a ZEISS LSM 800 laser scanning microscope (Bio-Rad).

Tumor tissues were harvested and fixed overnight in 4% paraformaldehyde and paraffin embedded. Sections (4 mm thick) were cut using a rotary microtome. The section slides were dipped in xylene and then 100%, 90%, 80%, and 70% ethanol solutions, respectively, and washed in running tap water for rehydration. For immunofluorescence, sections were subjected to antigen retrieval in 10 mM sodium citrate buffer (pH 6.0) for 20 minutes. The tissue sections were then incubated with 3% H_2_O_2_ for 15 minutes and blocked with 5% BSA in PBST (PBS with 0.2% Triton X-100 for 1 hour at room temperature. The slides were then incubated with CD8 (1:200; # NB200-578, Novus Biologicals, Centennial, CO, USA) and Granzyme B (1:200; # ab255598, Abcam, Cambridge, UK) overnight at 4 °C and then visualized with Alexa Fluor 488-conjugated goat anti-rat IgG (H+L) secondary antibody (1:400; # A-11006, Thermo Fisher Scientific, Waltham, MA, USA) and Alexa Fluor 594-conjugated goat anti-rabbit IgG (H+L) secondary antibody (1:400; # A-21244, Thermo Fisher Scientific, Waltham, MA, USA). Each slide was mounted in Vectashield mounting medium (Vector Laboratories, Burlingame, CA, USA) with DAPI to visualize the nuclei. The tissues were examined using an LSM 800 confocal laser-scanning microscope (Carl Zeiss, Jena, Germany).

For immunohistochemistry, the sections were incubated overnight at 4 °C with anti-CD206 antibody (1:200; Abcam, #ab64693, Cambridge, UK). The following day, the sections were incubated with an HRP-conjugated goat anti-rabbit IgG secondary antibody (1:400; Solarbio, Beijing, China) for 1 h at room temperature and developed using DAB substrate (Invitrogen). Sections were then counterstained with hematoxylin and examined under a microscope at 20× magnification in at least three randomly selected fields. All images were acquired using an LSM5 PASCAL microscope (Carl Zeiss, Jena, Germany), and total staining intensity was quantified using ImageJ software (NIH) (27).

### Co-culture of RAW264.7-derived macrophage and CD8^+^ T cells

CD8^+^ T cells were isolated from the spleens of 6-week-old C57BL/6 mice using a CD8a^+^ T Cell Isolation Kit (Miltenyi Biotec, Cat. No. 130-104-075) according to the manufacturer’s instructions. For T cell activation, isolated CD8^+^ T cells were cultured in plates coated with anti-CD3 antibody (5 μg/mL; BD biosciences, Cat. No. 553057) and supplemented with soluble anti-CD28 antibody (1 μg/mL) for 24 h. For the co-culture experiment, M2-polarized RAW264.7 macrophages were co-cultured with CD8^+^ T cells and treated with TAMpep-IP (0.5 μM). Macrophages and CD8^+^ T cells were co-cultured at a ratio of 1:5. After 48 h of co-culture, cells were harvested and stained with fluorochrome-conjugated antibodies for flow cytometric analysis of CD45, CD8, PD-1, and Granzyme B (28).

### Statistics

All data were analyzed using Prism 8.0.1 software (GraphPad Software Inc., San Diego, CA, USA) and are presented as mean ± standard error of the mean (SEM). Normality was assessed using the built-in normality test in GraphPad Prism. For statistical comparisons, an unpaired two-tailed Student’s t-test was used for two-group comparisons. For multiple group comparisons, one-way ANOVA followed by the Newman–Keuls *post hoc* test, or two-way ANOVA followed by Bonferroni correction, was applied as appropriate. p < 0.05 was considered statistically significant.

## Result

### TAMpep-IP reduces phosphorylation of STAT6 in M2 macrophages

**Figure 1:**
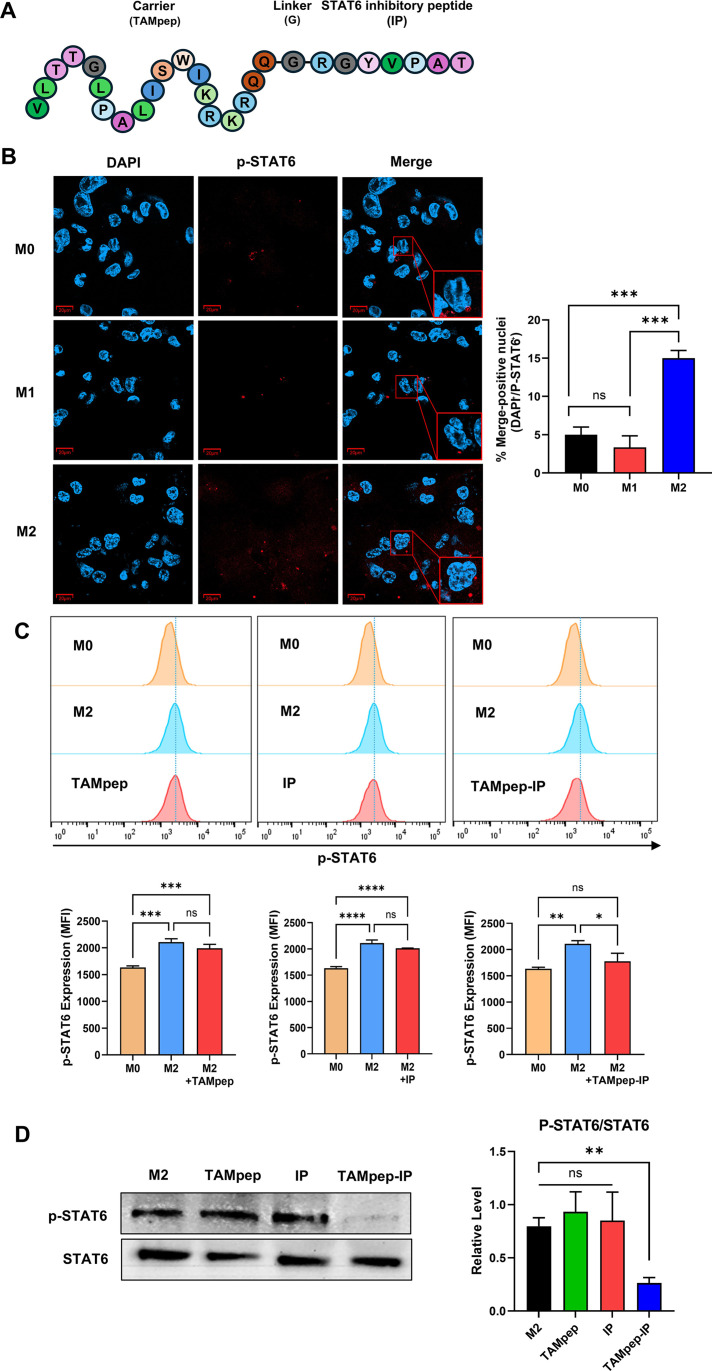
TAMpep-IP reduces phosphorylation of STAT6 in M2 macrophages. **(A)** Schematic structure of TAMpep-IP composed of an M2 macrophage-homing peptide (TAMpep), a cleavable linker, and a STAT6-inhibitory peptide (IP). **(B)** THP-1 cells were differentiated into M0, M1, or M2 macrophages and stained for phosphorylated STAT6 (p-STAT6; red). Nuclear staining was performed with DAPI (blue). Immunofluorescence microscopy revealed elevated nuclear p-STAT6 in M2 macrophages compared to M0 and M1. Representative confocal images were acquired using a 40× objective lens. Scale bars, 20 μm. **(C)** M0 and M2 macrophages were treated with TAMpep, IP, or TAMpep-IP (0.5 μM, 72 h). Flow cytometry was used to measure p-STAT6 expression levels (mean fluorescence intensity), showing that TAMpep-IP significantly reduced p-STAT6 in M2 macrophages. **(D)** Western blot analysis was performed to detect p-STAT6 and total STAT6 levels in M2 macrophages after administration with TAMpep, IP, or TAMpep-IP (0.5 μM, 72 h). TAMpep-IP effectively reduced p-STAT6. The experiment was performed in triplicate. All data are presented as mean ± SEM. *p<0.05, **p<0.01, ***p < 0.001 and ****p < 0.0001..

In TME, phosphorylation of STAT6 plays a pivotal role inducing M2 macrophage polarization, primarily through IL-4/IL-13-mediated signaling, thereby fostering an immunosuppressive environment that promotes cancer progression (29). TAMpep-IP is a peptide–drug conjugate (PDC) composed of an M2-homing peptide (TAMpep), a linker (G), and a STAT6-inhibitory peptide (IP), designed to suppress STAT6 in M2 TAMs. The structure of TAMpep-IP is shown in **Figure 1A**. To evaluate the effect of TAMpep-IP on STAT6 phosphorylation, we first examined p-STAT6 expression in THP-1-derived macrophages. Prior to this analysis, macrophage polarization was confirmed by flow cytometric of CD206 expression, demonstrating successful M2 polarization compared with M0 and M1 macrophages (**Supplementary Figure 3**). Immunofluorescence staining revealed that p-STAT6 levels increased in M2 macrophages compared to M0 and M1 macrophages (**Figure 1B**). TAMpep-IP reduced p-STAT6 expression level in M2 macrophages, whereas TAMpep or IP did not significantly affect p-STAT6 levels (**Figure 1C**). Consistently, TAMpep-IP decreased p-STAT6 levels in M2 macrophages compared with untreated controls (M2), while TAMpep or IP showed no inhibition (**Figure 1D**). Thus, these results suggest that TAMpep-IP selectively inhibits p-STAT6 expression in M2 macrophages.

### TAMpep-IP inhibits polarization of M2 macrophages

**Figure 2:**
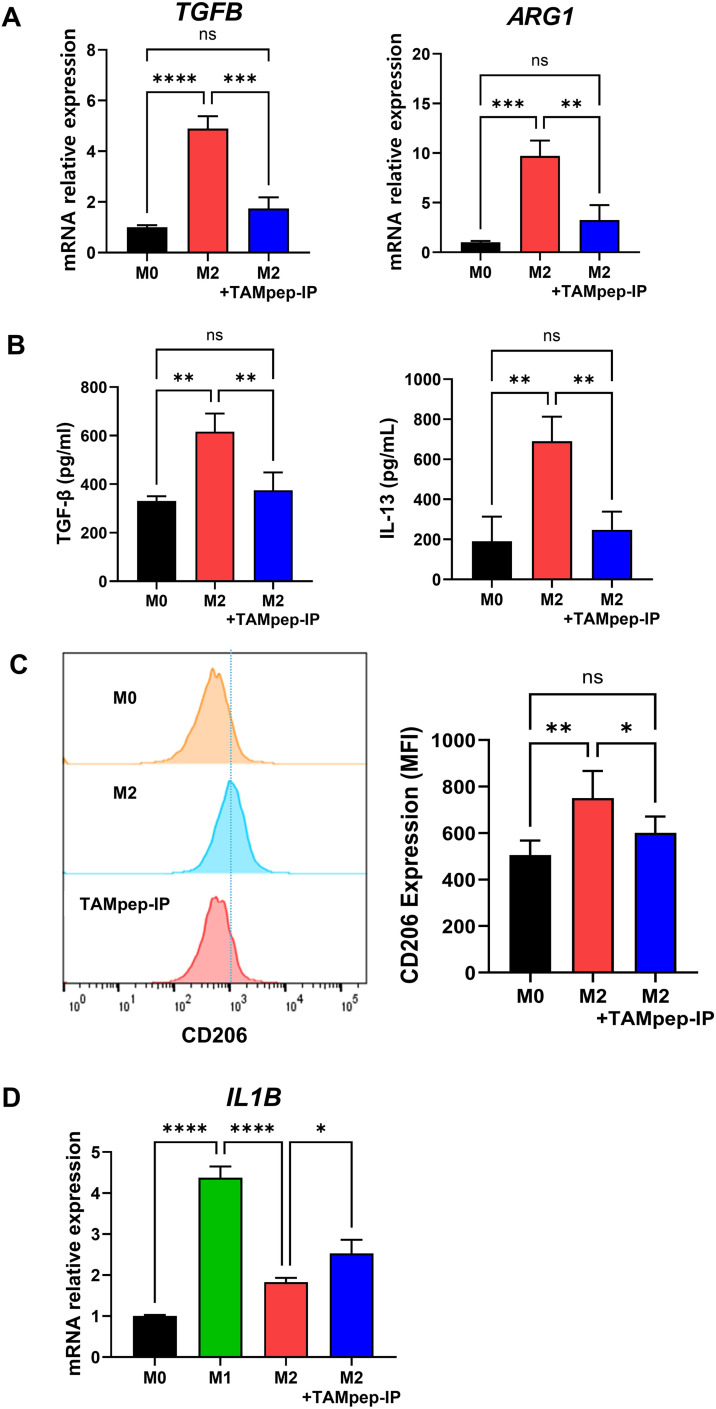
TAMpep-IP inhibits polarization of M2 macrophages. **(A)** THP-1 monocytes were differentiated into M0 and M2 macrophages, and M2 cells were treated with TAMpep-IP (0.5 μM, 72 h). Quantitative RT-PCR analysis showed that TAMpep-IP significantly reduced the mRNA expression of TGF-β and Arg-1, two markers associated with M2 polarization. **(B)** ELISA was performed to measure the levels of secreted TGF-β and IL-13 in the culture supernatant of M0, M2, and TAMpep-IP–treated M2 macrophages (72 h). TAMpep-IP markedly decreased secretion of both cytokines. **(C)** Flow cytometric analysis was used to assess CD206 surface expression in M0, M2, and TAMpep-IP–treated M2 macrophages (72 h). CD206 expression was significantly decreased in the TAMpep-IP group compared to untreated M2 macrophages. All data are presented as mean ± SEM. *p<0.05, **p<0.01, ***p<0.001, ****p < 0.0001. **(D)** The expression of IL-1β mRNA was analyzed by quantitative RT-PCR in M0, M1, and M2 macrophages after TAMpep-IP. TAMpep-IP significantly upregulated IL-1β expression in M2 macrophages, to levels comparable with M1-polarized cells.

To investigate the effect of STAT6 inhibition by TAMpep-IP on the expression of markers of M2 macrophages, we analyzed the expression of representative genes and proteins associated with M2 polarization, such as CD206, arginase 1, IL-13, and TGF-beta. mRNA levels of *TGFB* and *ARG1* were significantly upregulated in M2 macrophages compared to the M0 control, whereas TAMpep-IP treatment significantly reduced their expression (**Figure 2A**). Similarly, the production of TGF-β and IL-13, key immunosuppressive cytokines in M2 macrophages, was significantly reduced by TAMpep-IP (**Figure 2B**). Flow cytometric analysis revealed that TAMpep-IP reduced CD206 expression in M2 macrophages (**Figure 2C**). Furthermore, *IL1B* mRNA expression, which was relatively low in M2 macrophages compared to M1 macrophages, was increased by TAMpep-IP (**Figure 2D**). These results suggest that TAMpep-IP suppresses protein and gene expressions associated with M2 macrophage polarization while partially inducing inflammatory gene expression of IL-1β.

### TAMpep-IP suppresses tumor growth in the colon cancer model

**Figure 3:**
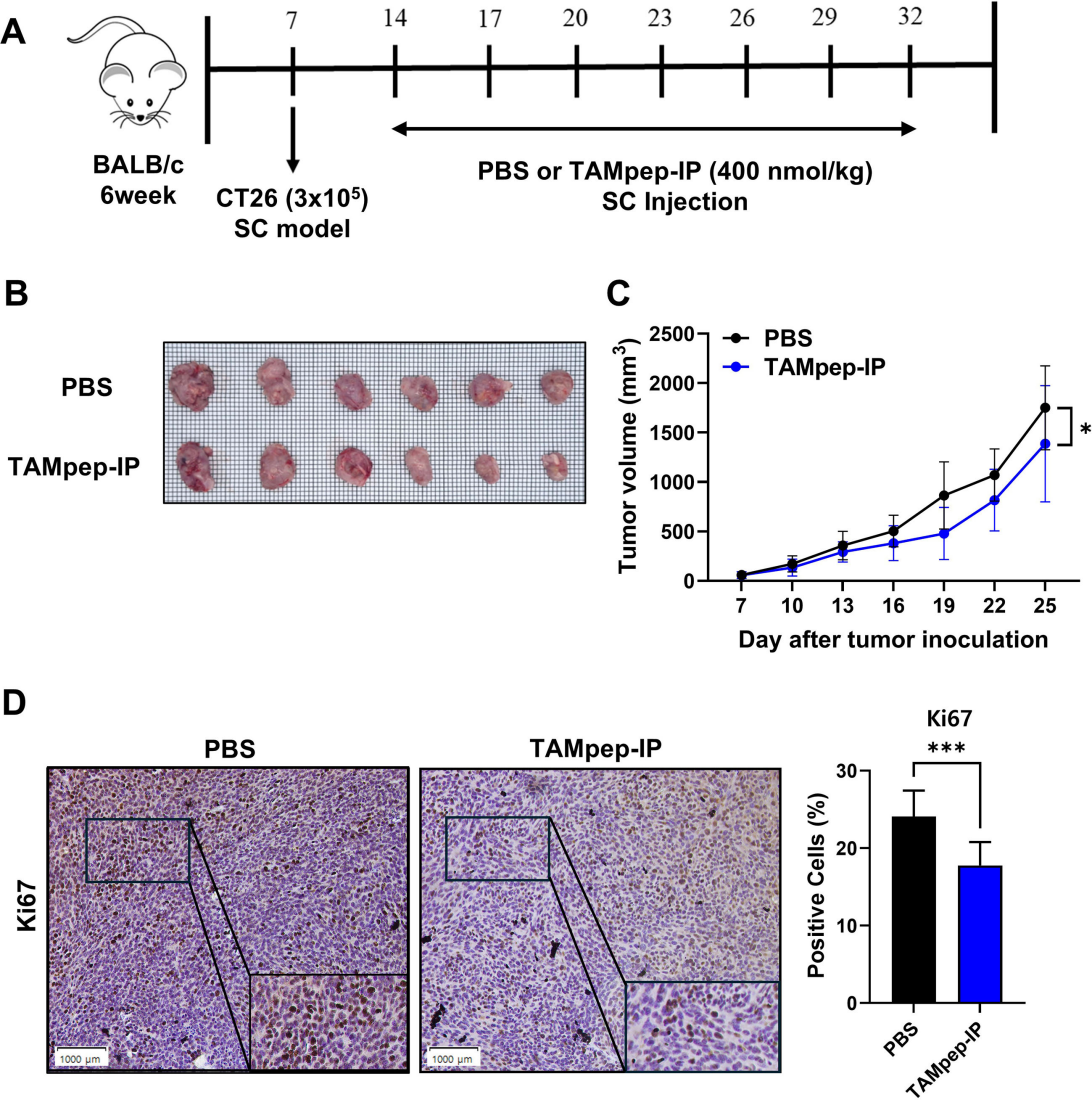
TAMpep-IP suppresses tumor growth in the colon cancer model. **(A)** BALB/c mice were subcutaneously inoculated with CT26 colon carcinoma cells (3 × 10^5^ cells per mouse). Starting on day 7 post-inoculation, TAMpep-IP (400 nmol/kg) was administered subcutaneously every three days for a total of seven doses. **(B)** Representative images of tumors excised at the experimental endpoint (day 25) showed visibly reduced tumor size in the TAMpep-IP–treated group compared to control. **(C)** Tumor volumes were measured every 3 days following tumor implantation. Mice treated with TAMpep-IP exhibited significantly reduced tumor growth relative to the control group (control: n = 6; TAMpep-IP: n = 6). **(D)** Tumor proliferation was evaluated by immunohistochemical staining of Ki-67 in tumor sections. Quantitative analysis showed a significantly lower proportion of Ki-67^+^ proliferating cells in TAMpep-IP–treated tumors. Representative immunohistochemistry images were acquired at ×100 magnification. Scale bar = 1000 μm. All data are presented as mean ± SEM. *p<0.05, ***p<0.001.

To evaluate the antitumor efficacy of TAMpep-IP in a colon cancer mouse model, CT26 cells (3 × 10^5^) were subcutaneously injected into BALB/c mice, and PBS or TAMpep-IP (400 nmol/kg) was administered every 3 days (**Figure 3A**). Tumor volume was significantly suppressed with TAMpep-IP compared to PBS, with a decrease observed from day 19 (**Figures 3B, C**). To investigate the effect of TAMpep-IP on tumor cell proliferation, tumor tissues were immunohistochemically stained for Ki67. As shown in **Figure 3D**, Ki67-positive cells were significantly reduced in the TAMpep-IP group compared to the PBS group. Thus, these results suggest that TAMpep-IP exerts antitumor effects by reducing tumor growth and inhibiting proliferation of tumor cells in a colorectal cancer model.

### TAMpep-IP reduces M2 macrophages in tumor tissues of colon cancer model

**Figure 4:**
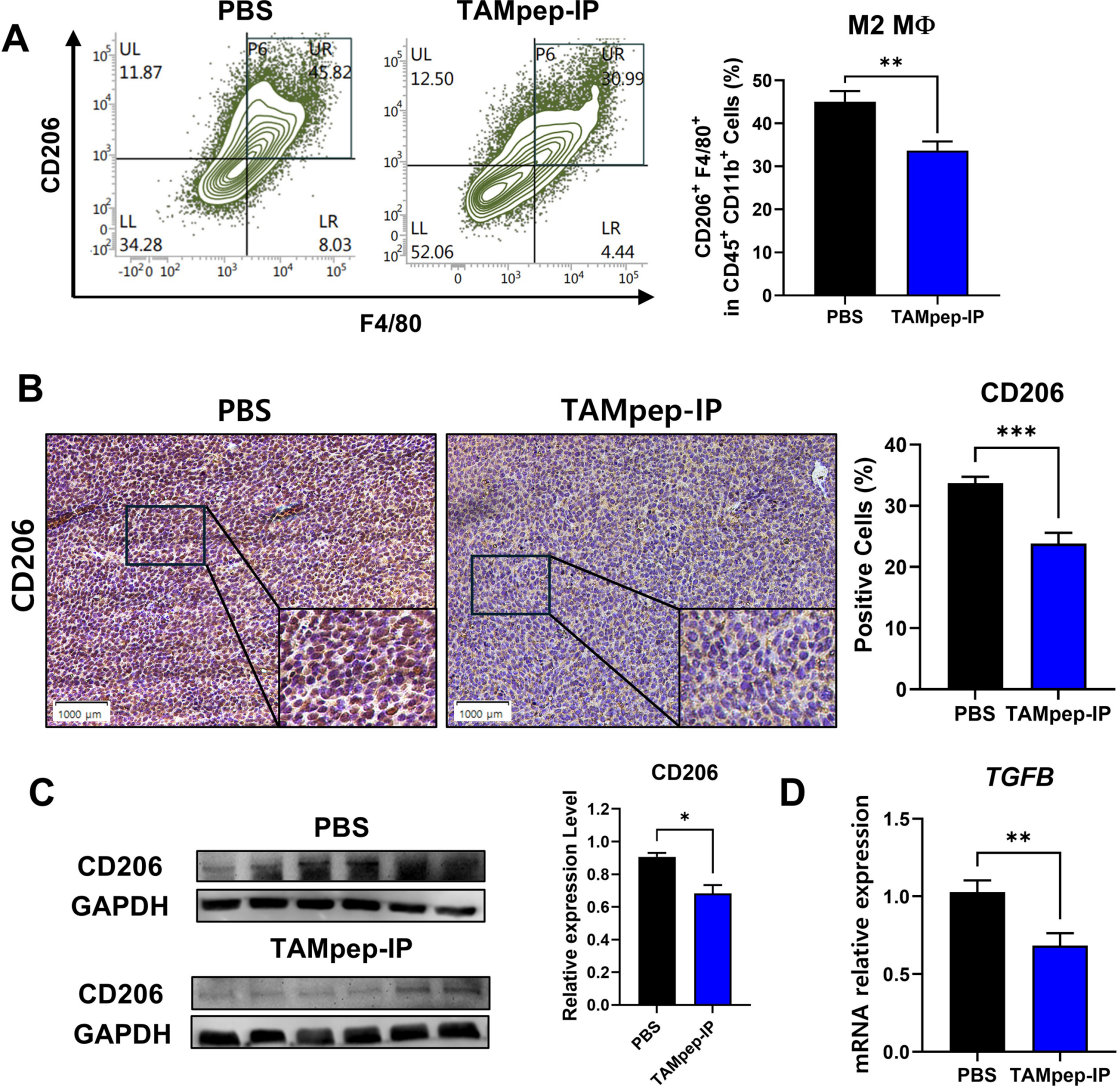
TAMpep-IP reduces M2 macrophages in tumor tissues of colon cancer model. **(A)** Tumor-infiltrating immune cells were isolated from CT26 tumors in control and TAMpep-IP–treated mice. Flow cytometry was used to identify CD206^+^ F4/80^+^ macrophages within the CD45^+^ CD11b^+^ population. TAMpep-IP significantly decreased the proportion of M2-like tumor-associated macrophages. **(B)** Quantitative RT-PCR analysis of tumor tissues revealed that TGF-β mRNA expression, a key M2-associated cytokine, was significantly reduced in TAMpep-IP–treated tumors compared to controls. **(C)** Western blot analysis of tumor showed a marked decrease in CD206 protein levels following TAMpep-IP, indicating effective suppression of M2 macrophage markers. **(D)** CD206^+^ macrophages were further visualized by immunohistochemical staining of tumor sections. ImageJ-based quantification confirmed a significant reduction in CD206^+^ area in TAMpep-IP–treated tumors. Representative immunohistochemistry images were acquired at ×100 magnification. Scale bar = 1000 μm. All data are presented as mean ± SEM. *p<0.05, **p<0.01, ***p<0.001.

To determine whether TAMpep-IP affects M2 TAMs in the tumor microenvironment, CD206^+^ macrophages obtained from tumor tissues were measured by flow cytometry. The gating strategy used to identify CD206^+^ tumor-associated macrophages is shown in **Supplementary Figure 4**. As shown in **Figure 4A**, TAMpep-IP significantly reduced the proportion of M2 macrophages compared to the PBS (**Figure 4A**). Next, we examined CD206-positive cells within tumor tissues using Immunohistochemical staining. Consistent with the flow cytometry results, TAMpep-IP significantly decreased CD206-positive cells compared to the PBS (**Figure 4B**). The CD206 protein expression in tumor tissues was reduced in TAMpep-IP group compared to the PBS group (**Figure 4C**). Furthermore, the mRNA level of *TGFB*, a key immunosuppressive cytokine associated with M2 macrophage activation, was significantly downregulated in TAMpep-IP group compared to the PBS group (**Figure 4D**). Therefore, these results suggest that TAMpep-IP alleviates immunosuppression by reducing the presence and activity of M2-like TAMs in the tumor microenvironment.

### TAMpep-IP enhances inflammatory cytokine expression and activated CD8^+^ T cells in tumor tissues of colon cancer model

**Figure 5:**
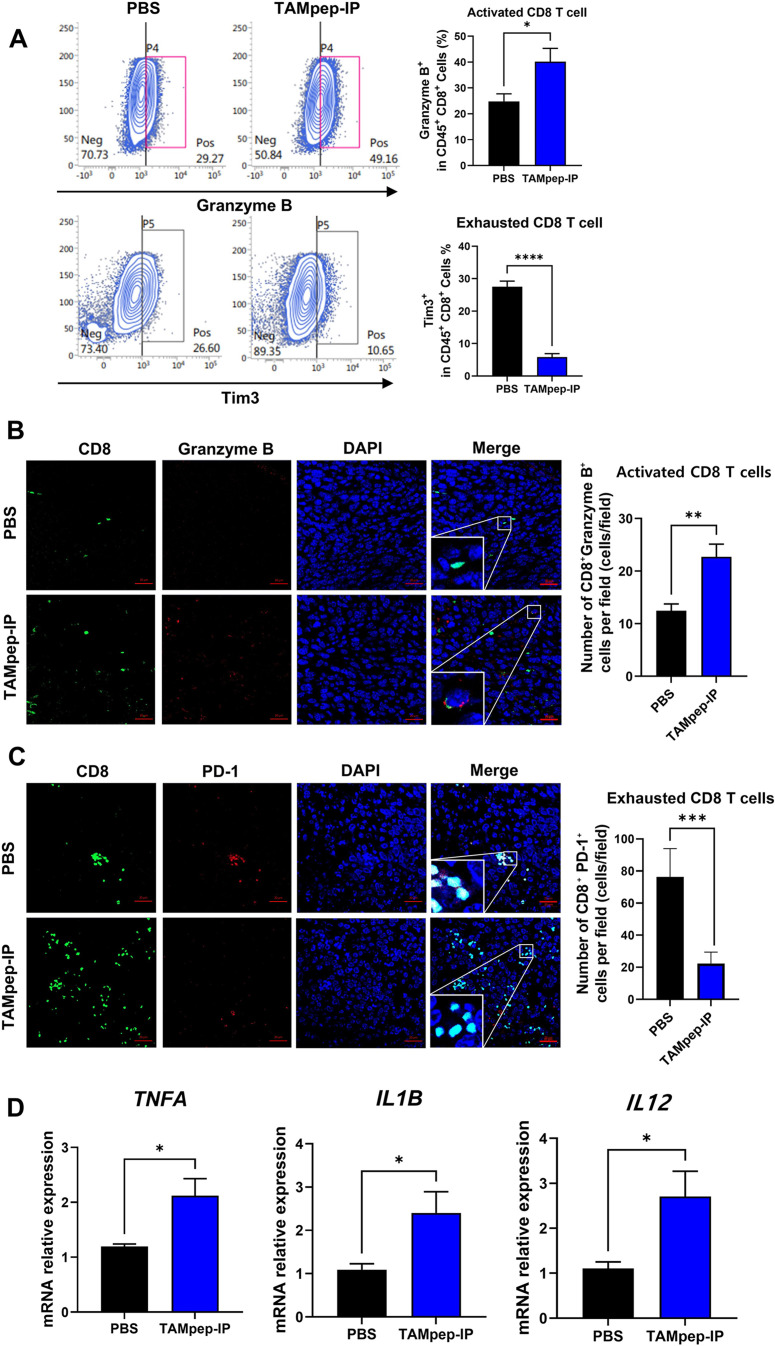
TAMpep-IP enhances inflammatory cytokine expression and activated CD8^+^ T cells in tumor tissues of colon cancer model. **(A)** Flow cytometry was used to evaluate CD8^+^ T cell function in CT26 tumor tissues from control and TAMpep-IP-treated mice. TAMpep-IP significantly increased the proportion of activated Granzyme B^+^ CD8^+^ T cells, while reducing the frequency of exhausted Tim-3^+^ CD8^+^ T cells, indicating enhanced cytotoxic T cell activity. **(B, C)** Confocal immunofluorescence analysis was performed on tumor sections stained with DAPI (nuclei), anti-CD8 (green), anti-Granzyme B (red), and anti-PD-1 (red). Activated CD8^+^ T cells were identified by co-localization of CD8 and Granzyme B, whereas exhausted CD8^+^ T cells were identified by co-localization of CD8 and PD-1. Quantification revealed a significant increase in intertumoral CD8^+^ Granzyme B^+^ T cells and a concomitant decrease in CD8^+^ PD-1^+^ exhausted T cells following TAMpep-IP. Representative confocal images were acquired using a 40× objective lens. Scale bar = 20 μm. **(D)** Quantitative RT-PCR analysis of CT26 tumor tissues showed significantly elevated mRNA levels of pro-inflammatory cytokines TNF-α, IL-1β, and IL-12 in TAMpep-IP–treated mice compared to controls, indicating induction of a pro-inflammatory tumor microenvironment. All data are presented as mean ± SEM. *p<0.05, **p<0.01, ***p<0.001, ****p < 0.0001.

To determine whether TAMpep-IP affects CD8^+^ T cell activation in the tumor microenvironment, we analyzed the proportion of activated (Granzyme B^+^) and exhausted (Tim3^+^) CD8^+^ T cells using flow cytometry. The gating strategies for flow cytometric analyses of CD8^+^ T-cell activation and exhaustion are shown in **Supplementary Figure 4**. TAMpep-IP significantly increased the proportion of Granzyme B^+^ CD8^+^ T cells compared with PBS and significantly decreased the proportion of Tim3^+^ CD8^+^ T cells (**Figure 5A**). Moreover, the number of CD8^+^ T cells co-expressing Granzyme B in tumor tissues was higher in the TAMpep-IP group than in the PBS group (**Figure 5B**). In contrast, the number of PD-1-expressing CD8^+^ T cells was significantly reduced in the TAMpep-IP group (**Figure 5C**). To evaluate the induction of inflammatory cytokine expression by TAMpep-IP, we measured *TNFA*, *IL1B*, and *IL12* mRNA levels in tumor tissues using qRT-PCR. TAMpep-IP significantly upregulated all three cytokines compared to PBS (**Figure 5D**). To further investigate whether TAMpep-IP modulated macrophages directly influence CD8^+^ T cell function, we performed co-culture assay using RAW 264.7-derived M2 macrophages and CD8^+^ T cells. TAMpep-IP significantly reduced PD-1 expression in naïve T cells and increased Granzyme B expression in activated CD8^+^ T cells (**Supplementary Figure 5**). Therefore, these results suggest that TAMpep-IP enhances antitumor immunity by promoting activation of CD8^+^ T cells, reducing exhaustion of CD8^+^ T cells, and increasing production of inflammatory cytokines in the tumor microenvironment.

## Discussion

In this study, we demonstrated that selective inhibition of STAT6 in M2 TAMs by TAMpep IP effectively reprogrammed the TME and suppressed tumor growth in a CRC model. TAMpep IP, a peptide drug conjugate composed of an M2 homing peptide (TAMpep) and a STAT6 inhibitory sequence (RGYVPAT), selectively reduced STAT6 phosphorylation in M2 macrophages while sparing M0 and M1 subtypes. The dose of TAMpep-IP used in this study, 400nmol/kg, was selected based on our preliminary dose-evaluation experiments shown in **Supplementary Figure 6**. Among the tested doses of 100, 200, and 400nmol/kg, 400nmol/kg produced the evident antitumor effect and reduction in the M2-like macrophage population, as assessed by tumor volume measurement and flow cytometric analysis. Importantly, a previous study demonstrated that the M2-homing peptide TAMpep used in this study associates with M2-like TAMs in tumor tissues, supporting the targeting specificity of TAMpep IP toward CD206^+^ macrophages (27). In the same study, cellular uptake/localization analysis using fluorescently labeled TAMpep further demonstrated the preferential localization of TAMpep in M2 macrophages. Although cellular uptake or localization of fluorescently labeled TAMpep-IP was not directly assessed in the present study, these previous findings provide evidence for using TAMpep as the M2-homing carrier peptide to deliver the STAT6 inhibitory peptide to M2 macrophages. Nevertheless, direct visualization of TAMpep-IP uptake and intracellular localization will be required in future studies to further validate its targeting specificity. Consequently, TAMpep IP downregulated M2-associated markers, including CD206, Arg1, IL-13, and TGF-β, while increasing pro-inflammatory mediators such as IL-1β. Importantly, this molecular reprogramming translated into marked antitumor efficacy *in vivo*, characterized by reduced tumor burden, decreased Ki-67 proliferation, and enhanced CD8^+^ T cell activation with diminished exhaustion. These findings collectively highlight the functional significance of the STAT6–M2 axis in maintaining an immunosuppressive TME and provide the possibility that its targeted disruption can restore antitumor immune balance in CRC.

STAT6 is a key transcription factor activated downstream of IL-4 and IL-13 and plays a central role in macrophage polarization toward the M2 phenotype (30). Activated STAT6 translocates to the nucleus to induce transcription of M2-associated genes such as *Arg1*, *CD206*, and *IL10*, thereby promoting tissue remodeling and tumor progression (29, 31). Inhibition of STAT6 signaling, either by genetic knockdown or pharmacologic blockade, has been shown to suppress M2 polarization and enhance antitumor immunity in multiple cancer models, including colon, lung, and breast cancer (32, 33). In particular, McCusker et al. demonstrated that a cell-penetrating STAT6 inhibitory peptide effectively blocked STAT6 phosphorylation at Tyr641 and reduced downstream expression of STAT6-dependent cytokines, providing direct evidence for the feasibility of targeting STAT6 (26). In this study, we observed that phosphorylation of STAT6 was markedly increased in IL-4/IL-13-polarized M2 macrophages, and showed that TAMpep-IP, a conjugate of STAT6 inhibitory peptide (IP) and the M2-homing peptide TAMpep, selectively reduced p-STAT6 expression in M2 macrophages, whereas TAMpep or IP alone had no effect.

Within the TME, M2 TAMs constitute a major immunosuppressive population that promotes tumor progression, angiogenesis, and metastasis (34, 35). Unlike M1 macrophages, which mediate pro-inflammatory and antitumor responses, M2 TAMs secrete immunosuppressive cytokines such as TGF-β and IL-10, thereby fostering an environment conducive to tumor growth and immune evasion (36). Consistent with this, numerous studies have reported that high M2 TAM infiltration correlates with poor prognosis in colorectal, breast, and pancreatic cancers (30). Thus, targeting M2 TAMs or reprogramming them toward a pro-inflammatory M1-like phenotype has emerged as a promising therapeutic strategy to overcome immune suppression in solid tumors (19, 37). In our study, TAMpep-IP treatment markedly reduced the expression of M2-associated markers, including CD206, Arg-1, and TGF-β, indicating suppression of M2 polarization. Importantly, TAMpep-IP also upregulated *IL1B* expression, a gene typically associated with M1 macrophage activation and inflammatory cytokine production. In particular, IL-1β is known to be a key mediator that promotes antitumor immune responses through the recruitment and activation of CD8^+^ T cells (38, 39). Similar results have been observed in studies where inhibition of M2-associated signaling pathways, such as STAT6, PI3Kγ, or CSF-1R, induces macrophage repolarization, thereby increasing T cell infiltration and tumor regression (24, 40).

In colon cancer, accumulating evidence indicates that M2 TAMs play a crucial role in promoting tumor initiation, growth, and metastasis (41, 42). Clinical studies have shown that a high density of CD163^+^ or CD206^+^ M2 macrophages in CRC tissues correlates with advanced tumor stage, increased angiogenesis, and poor patient survival (43). Functionally, M2 TAMs secrete immunosuppressive cytokines such as TGF-β, IL-10, and VEGF, which inhibit CD8^+^ T cell cytotoxicity and promote tumor cell proliferation (44). Conversely, inhibition or depletion of M2 TAMs has been shown to suppress tumor progression in CRC models (40). For example, pharmacologic blockade of CSF-1R signaling significantly reduced M2 macrophage infiltration and tumor growth (45). while inhibition of STAT6 signaling reprogrammed TAMs toward a pro-inflammatory M1 phenotype, leading to tumor regression and enhanced antitumor immunity (46). M2 TAMs represent a dominant immunoregulatory component that promotes tumor growth, angiogenesis, and metastasis, and their abundance generally correlates with poor prognosis (47). Consistent with these findings, our study demonstrated that TAMpep-IP effectively suppressed tumor growth and proliferation in a CRC model.

CD8^+^ T cells are essential mediators of antitumor immunity, and their cytotoxic potential is largely defined by the expression of effector molecules such as Granzyme B and Perforin. However, persistent antigen stimulation and suppressive cytokine signaling in the TME induce an exhausted phenotype characterized by high expression of inhibitory receptors such as PD-1, TIM-3, and LAG-3, leading to functional inactivation (48, 49). Studies have demonstrated that M2 TAMs inhibit T cell-mediated antitumor immunity through both soluble factors and cell-cell interactions (50, 51). High infiltration of M2 macrophages in colorectal and hepatocellular carcinomas has been associated with increased numbers of PD-1^+^ exhausted CD8^+^ T cells and poor clinical outcome (52). Moreover, depletion or reprogramming of M2 TAMs has been reported to restore CD8^+^ T cell effector function, characterized by enhanced Granzyme B expression and decreased PD-1 and TIM-3 positivity (53, 54). In our study, TAMpep-IP resulted in a marked increase in Granzyme B^+^ CD8^+^ T cells and a reduction in PD-1^+^ and TIM-3^+^ exhausted CD8^+^ T cells within tumor tissues. Moreover, the increase in inflammatory cytokines (TNF-α, IL-1β, IL-12) observed in our study further supports that TAMpep-IP reshapes the TME into an immunostimulatory environment favorable for potent T cell cytotoxicity. Although TAMpep-IP showed clear antitumor efficacy in the present CRC model, its pharmacokinetic profile, serum stability, half-life, and biodistribution were not evaluated in this study. This represents an important limitation, as peptide-based therapeutics may be susceptible to proteolytic degradation under physiological conditions, which could affect the stability, bioavailability, and duration of action of TAMpep-IP. Future studies will be needed to assess the serum stability and pharmacokinetic properties of TAMpep-IP and to optimize its formulation for improved translational applicability.

## Conclusion

In this study, we developed a peptide drug conjugate, TAMpep-IP, designed to inhibit STAT6 signaling in M2 TAMs. Our results demonstrated that TAMpep-IP effectively suppressed STAT6 activation in M2 macrophages, thereby inhibiting their polarization. Furthermore, TAMpep-IP treatment reduced the accumulation of M2 TAMs within tumor tissues of CRC model, leading to decreased tumor growth and enhanced cytotoxic CD8^+^ T-cell activity. This selective approach represents a promising therapeutic strategy to overcome M2 TAM-mediated immunosuppression and may improve the efficacy of existing immunotherapies.

## Data Availability

The original contributions presented in the study are included in the article/**Supplementary Material**. Further inquiries can be directed to the corresponding authors.
