# Supplementary material for: STAT6 inhibition of M2 macrophages suppresses tumor growth by modulating the tumor microenvironment in colon cancer model
Source: Front Immunol. 2026 Jun 3;17:1733991. doi: 10.3389/fimmu.2026.1733991 (PMC13272300; doi:10.3389/fimmu.2026.1733991)
Supplement: Supplementary file 1 [file Table1.docx]

**Supplementary Table 1. qRT-PCR Human primers for analysis**

| **Gene** | **F/R** | **Sequence** | **Reference** |
| --- | --- | --- | --- |
| **GAPDH** | **F** | 5’-GGG AGC CAA AAG GGT CAT CA-3′ | NM_001256799.3 |
|  | **R** | 5’-TGA TGG CAT GGA CTG TGG TC-3′ |  |
| **TGF-β** | **F** | 5’-GGA CTC TGA TAA CAC CCA TT-3’ | NM_000660.7 |
|  | **R** | 5’-TCA AGT AAT CCT CCC ATC TCT T-3’ |  |
| **Arginase-1** | **F** | 5’-CCT TTG CTG ACA TCC CTA AT-3’ | NM_000045.4 |
|  | **R** | 5’-CTT CCG TTC TTC TTG ACT TCT-3’ |  |
| **IL-1β** | **F** | 5’-TTC TCT TCA GCC AAT CTT CAT-3’ | NM_000576.3 |
|  | **R** | 5’-AGG AGC ACT TCA TCT GTT TAG-3’ |  |

**Supplementary Table 2. qRT-PCR Mouse primers for analysis**

| **Gene** | **F/R** | **Sequence** | **Reference** |
| --- | --- | --- | --- |
| **GAPDH** | **F** | 5’-GAG AAA CCT GCC AAG TAT GA-3’ | NM_001289726.2 |
|  | **R** | 5’-CTG TTG CTG TAG CCG TAT T-3’ |  |
| **TGF-β** | **F** | 5’-GTG ACA GCA AAG ATA ACA AAC TC-3’ | NM_011577.2 |
|  | **R** | 5’-AAC CCA GGT CCT TCC TAA A-3’. |  |
| **TNF-α** | **F** | 5’-CCA CCA TCA AGG ACT CAA AT-3’ | NM_001278601.1 |
|  | **R** | 5’-CTC AGG GAA GAA TCT GGA AAG-3’ |  |
| **IL-1β** | **F** | 5’-ATC CCA AGC AAT ACC CAA AG-3’ | NM_008361.4 |
|  | **R** | 5’-TAG AAA CAG TCC AGC CCA TA-3’ |  |
| **IL-12** | **F** | 5’-GAT GTG TCC TCA GAA GCT AAC-3’ | NM_001303244.1 |
|  | **R** | 5’-CAG TCC ACC TCT ACA ACA TAA A-3’ |  |


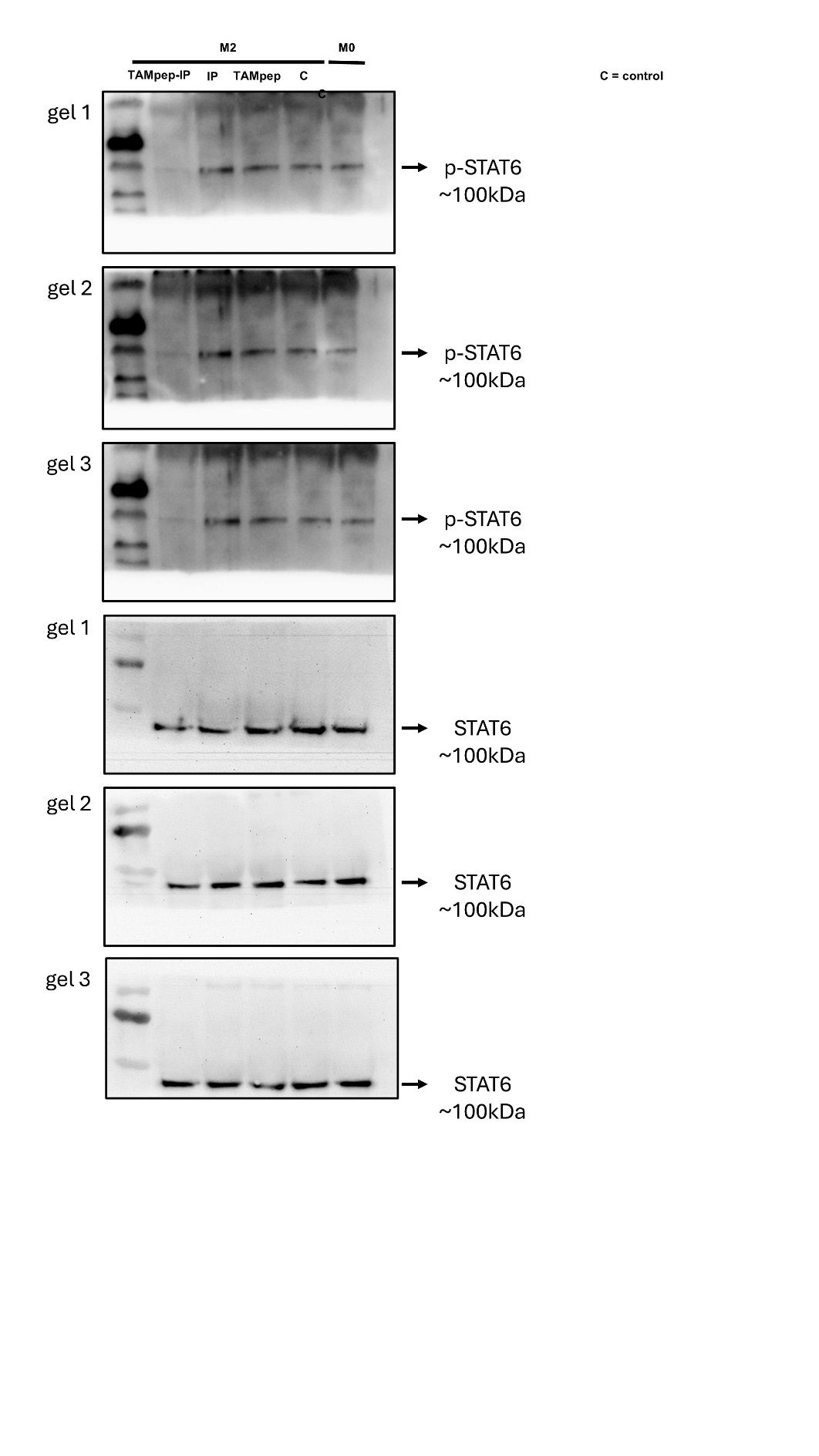


**Supplementary Figure 1. Western blot gel image**

Western blot analysis was performed to detect p-STAT6 and total STAT6 levels in M0, M2 and M2 macrophages after administration with TAMpep, IP, or TAMpep-IP (0.5 μM, 72 h). C = control


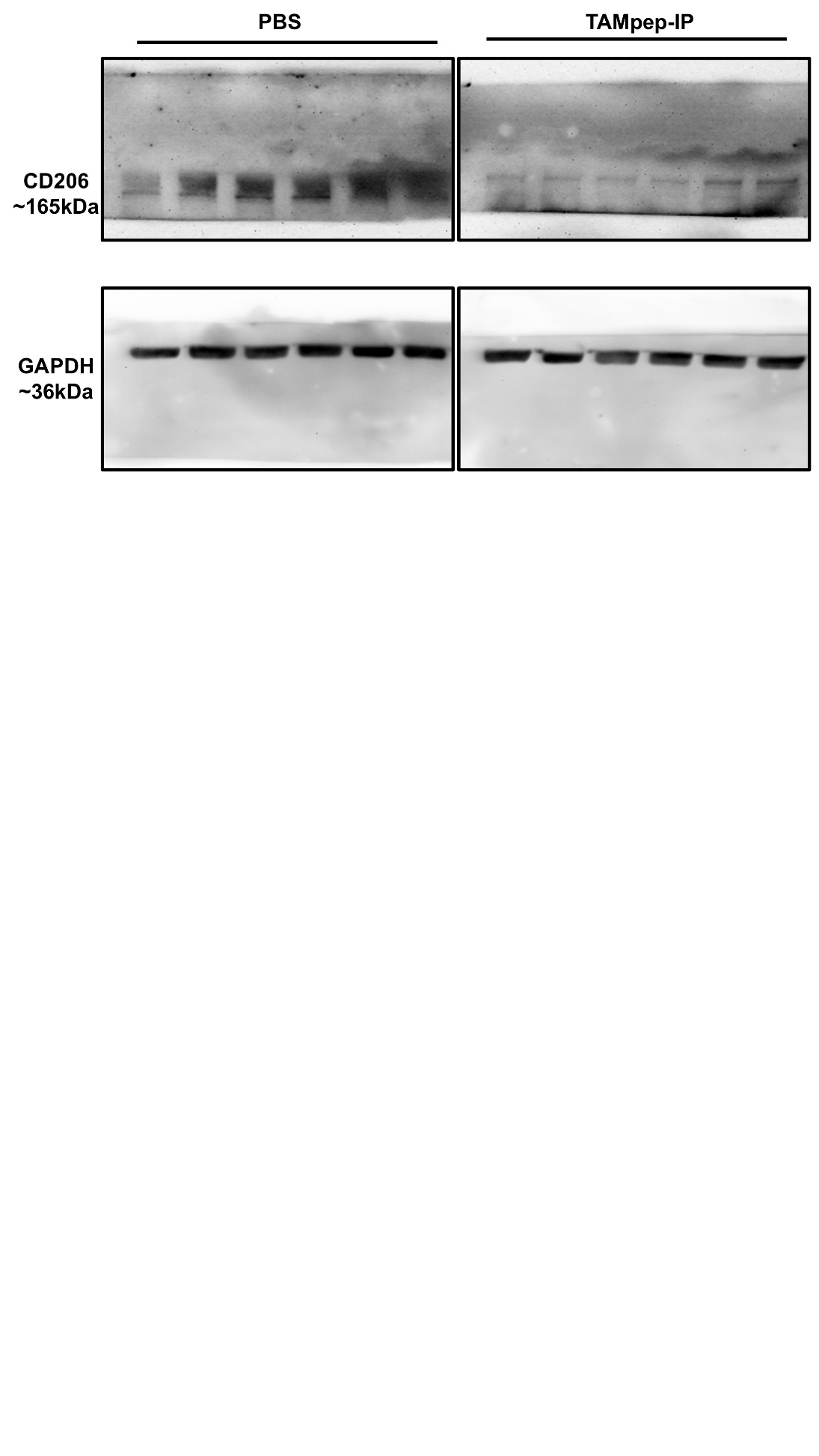


**Supplementary Figure 2. Western blot gel image**

Western blot analysis was performed to detect CD206 protein in the tumor tissues of colon cancer mouse model.


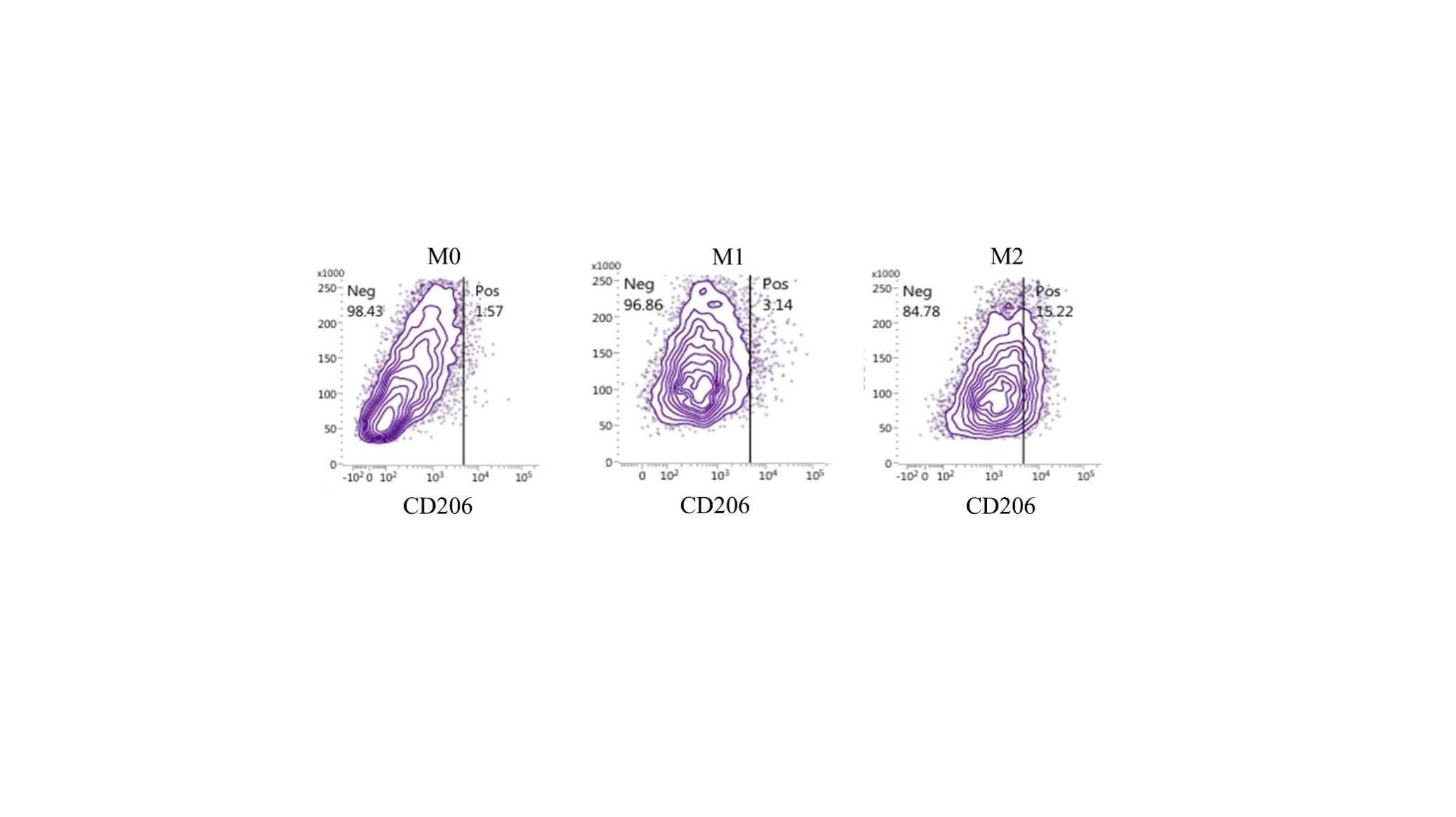


**Supplementary Figure 3. Flow cytometric analysis of CD206 expression in polarized macrophages.**

THP-1–derived macrophages were polarized into M0, M1, or M2 macrophages as described in the methods. Representative flow cytometry plots show surface expression of the M2 macrophage marker CD206 in each macrophage. CD206 expressions were markedly increased in M2 macrophages compared with M0 and M1 macrophages.


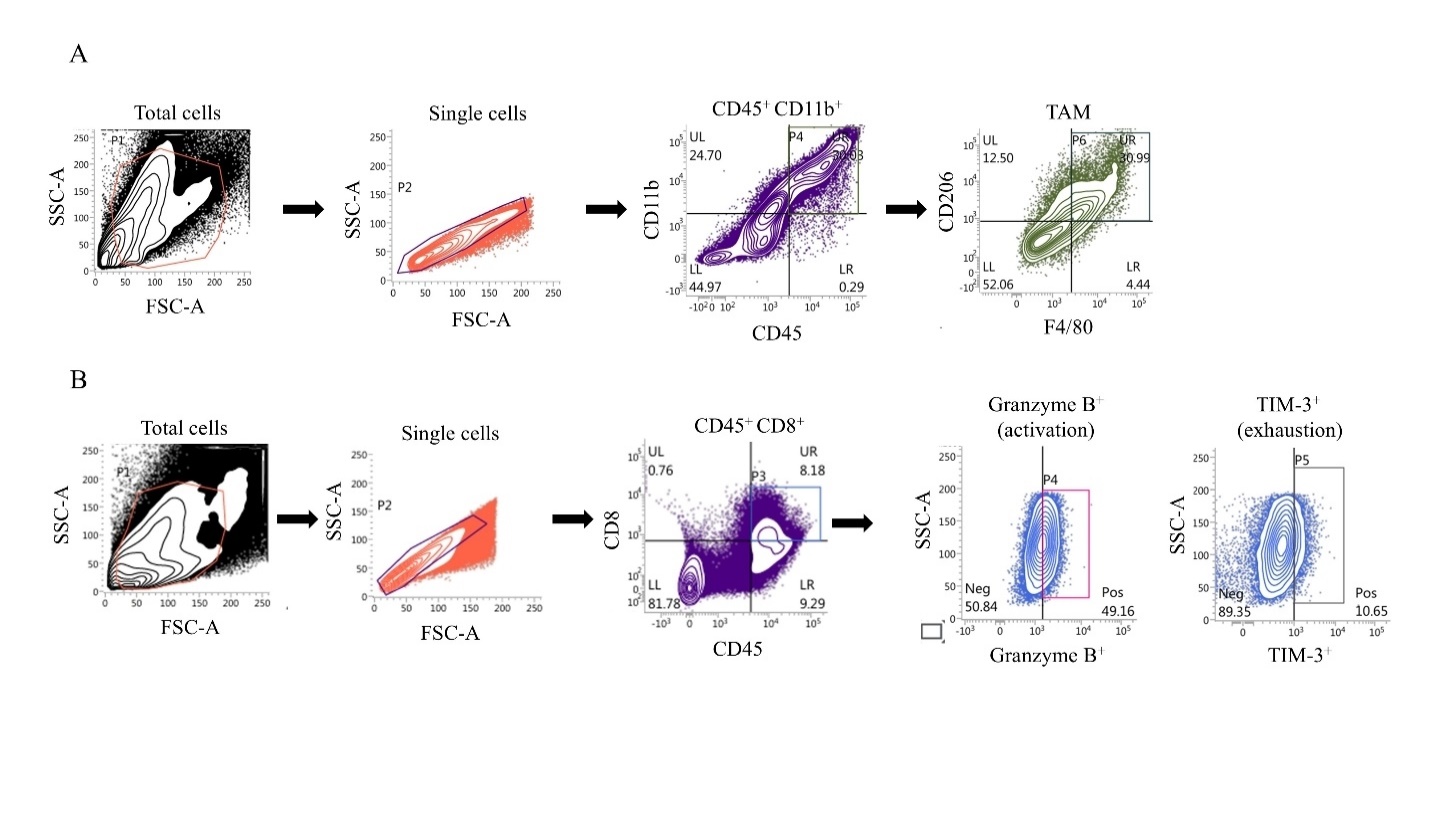


**Supplementary Figure 4. Gating strategies for TAMs and CD8⁺ T-cell populations**

Representative flow cytometry plots showing the sequential gating strategy used to identify CD206⁺ tumor-associated macrophages (A) and activated or exhausted CD8⁺ T-cell populations (B) from tumor tissues.


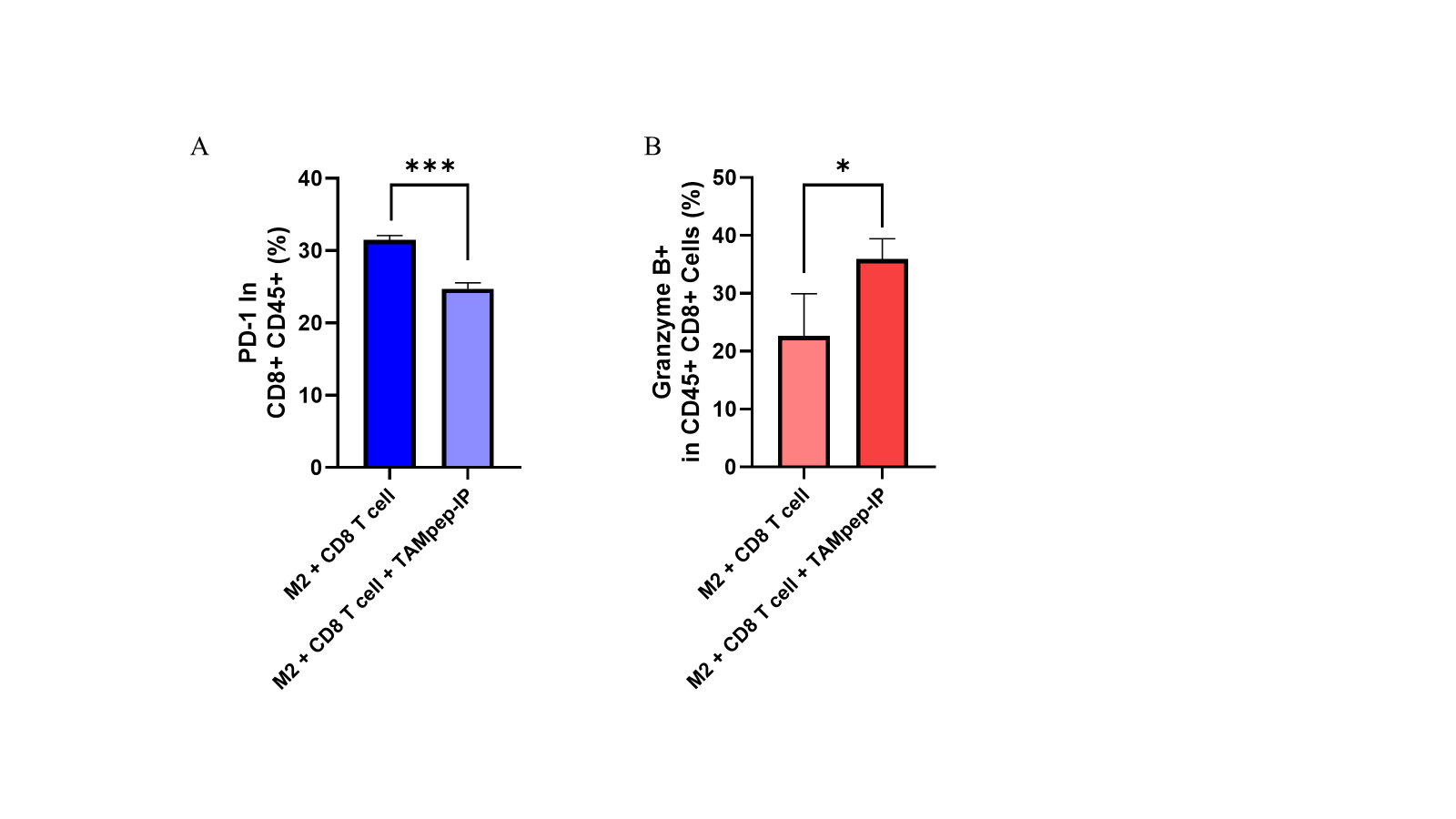


**Supplementary Figure 5. TAMpep-IP-treated M2 macrophages regulate CD8 T cell exhaustion and cytotoxic activation in a RAW264.7-derived macrophage-T cell co-culture system.**

A) Percentage of PD-1⁺ cells among CD45⁺CD8⁺ T cells co-cultured with RAW264.7-derived M2 macrophages and naive CD8 T cell in the presence or absence of TAMpep-IP(0.5 μM, 48h), analyzed by flow cytometry. (B) Percentage of Granzyme B⁺ cells among CD45⁺CD8⁺ T cells co-cultured with RAW264.7-derived M2 macrophages and activated CD8 T cell in the presence or absence of TAMpep-IP(0.5μM, 48h), determined by flow cytometry.


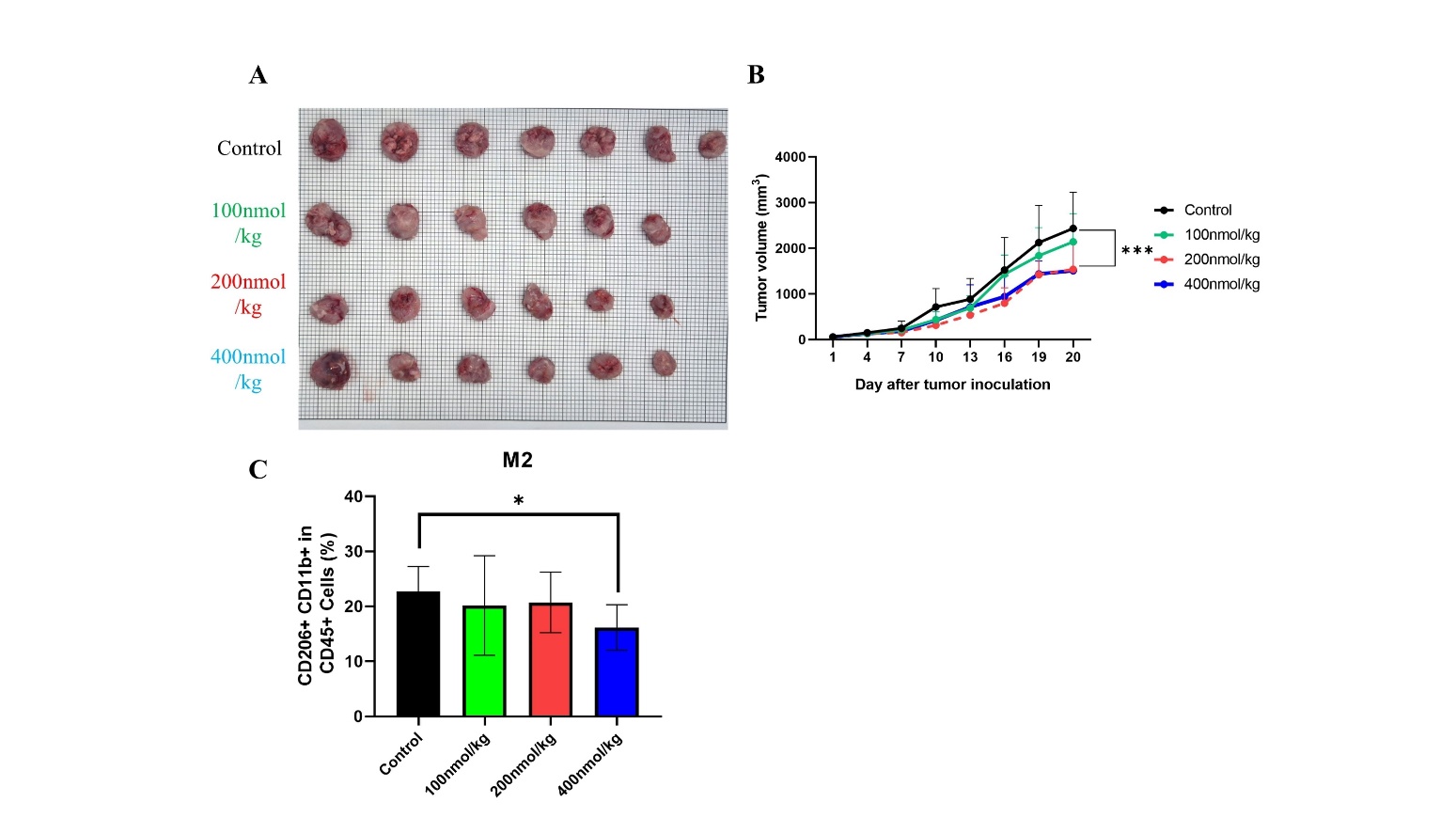


**Supplementary Figure 6. Dose-evaluation of TAMpep-IP in a colon cancer subcutaneous mouse model.**

A) Representative images of tumors from CT26 tumor-bearing mic treated with PBS control, TAMpep-IP at 100, 200, and 400nmol/kg. B) Tumor volume changes during the treatment peroid. C) Flow cytometric analyssis of M2-like macrophages, defined as CD206⁺ CD11b⁺ cells within CD45⁺ immune cells, in tumor tissues.
